# Analysis of Gene-Gene Interactions among Common Variants in Candidate Cardiovascular Genes in Coronary Artery Disease

**DOI:** 10.1371/journal.pone.0117684

**Published:** 2015-02-06

**Authors:** Muntaser D. Musameh, William Y. S. Wang, Christopher P. Nelson, Carla Lluís-Ganella, Radoslaw Debiec, Isaac Subirana, Roberto Elosua, Anthony J. Balmforth, Stephen G. Ball, Alistair S. Hall, Sekar Kathiresan, John R. Thompson, Gavin Lucas, Nilesh J. Samani, Maciej Tomaszewski

**Affiliations:** 1 Department of Cardiovascular Sciences, University of Leicester, Glenfield Hospital, Leicester, United Kingdom; 2 NIHR Leicester Cardiovascular Biomedical Research Unit, Glenfield Hospital, Leicester, United Kingdom; 3 School of Medicine, University of Queensland, Brisbane, Queensland, Australia; 4 Cardiovascular Epidemiology and Genetics, IMIM, Barcelona, Spain; 5 Epidemiology and Public Health Network (CIBERESP), Barcelona, Spain; 6 University of Leeds, MCRC, Leeds Institute of Genetics, Health and Therapeutics, Leeds, United Kingdom; 7 Division of Epidemiology, LIGHT, School of Medicine, University of Leeds, Leeds, United Kingdom; 8 The Broad Institute of MIT and Harvard, Cambridge, Massachusetts, United States of America; University Heart Center, GERMANY

## Abstract

**Objective:**

Only a small fraction of coronary artery disease (CAD) heritability has been explained by common variants identified to date. Interactions between genes of importance to cardiovascular regulation may account for some of the missing heritability of CAD. This study aimed to investigate the role of gene-gene interactions in common variants in candidate cardiovascular genes in CAD.

**Approach and Results:**

2,101 patients with CAD from the British Heart Foundation Family Heart Study and 2,426 CAD-free controls were included in the discovery cohort. All subjects were genotyped with the Illumina HumanCVD BeadChip enriched for genes and pathways relevant to the cardiovascular system and disease. The primary analysis in the discovery cohort examined pairwise interactions among 913 common (minor allele frequency >0.1) independent single nucleotide polymorphisms (SNPs) with at least nominal association with CAD in single locus analysis. A secondary exploratory interaction analysis was performed among all 11,332 independent common SNPs surviving quality control criteria. Replication analyses were conducted in 2,967 patients and 3,075 controls from the Myocardial Infarction Genetics Consortium. None of the interactions amongst 913 SNPs analysed in the primary analysis was statistically significant after correction for multiple testing (required P<1.2x10^-7^). Similarly, none of the pairwise gene-gene interactions in the secondary analysis reached statistical significance after correction for multiple testing (required P = 7.8x10^-10^). None of 36 suggestive interactions from the primary analysis or 31 interactions from the secondary analysis was significant in the replication cohort. Our study had 80% power to detect odds ratios > 1.7 for common variants in the primary analysis.

**Conclusions:**

Moderately large additive interactions between common SNPs in genes relevant to cardiovascular disease do not appear to play a major role in genetic predisposition to CAD. The role of genetic interactions amongst less common SNPs and with medium and small magnitude effects remain to be investigated.

## Introduction

The past few years have seen a major success in identifying common alleles associated with coronary artery disease (CAD) risk through genome wide association studies (GWAS) [1,2]. Interestingly, the identified variants to date explain only about 10% of the heritable component of inter-individual variation in CAD risk [1]. Amongst possible mechanisms that may account for some of the missing heritability, gene-gene interactions are intuitively attractive [3]. The biological mechanisms mediating genetic effects usually involve several genes. Strategies investigating such genes individually, risk overlooking their effects unless they take into account their possible interactions [3]. Furthermore, uncovering gene-gene interactions may yield key information to help understand the biological mechanisms underlying complex traits and diseases [4]. The role of gene-gene interactions has been systematically examined only in a number of complex human diseases [5,6] and only a Few studies have examined gene-gene interactions in CAD mainly through candidate gene approaches [7–9].

Analysis of genetic interactions poses a significant computational challenge and results in heavy penalty for multiple testing. Full two-way interaction analysis of 550,000 SNPs from 1200 individuals may take up to 120 days to complete when performed on a single 3GHz computer [10]. Furthermore, unlike conventional single SNP-based GWAS, there is no widely accepted significance threshold for genome-wide interaction analysis. Becker et al. [11] suggested an uncorrected P-value of 1.0x10^-12^ as a cut-off for statistical significance in an allelic interaction test conducted on 500,000 SNPs assuming type 1 error at 0.05.

Given these challenges inherent to genome-wide interaction analysis, prioritisation of the tested SNPs to enhance the chances to detect genuine interactions has considerable appeal. Biological plausibility, involvement in specific biological pathways and nominal level of statistical significance at an individual SNP level are among the commonly proposed approaches to reduce the size of the tested SNP population and therefore minimise the penalty for multiple testing [10,12].

In this study we selected common SNPs from a gene-centric array (Illumina HumanCVD BeadChip—IBC 50K array) enriched for genes and pathways relevant to the cardiovascular system and cardiovascular disease [13]. First, we conducted gene-gene interaction analysis among a set of independent SNPs with a minor allele frequency of ≥10% and at least nominal single marker association with CAD. This method has the potential advantage of testing interactions with higher prior probability of disease association, as well as testing a smaller set of makers with potential interactions which requires less correction for multiple testing. We then conducted an additional more exploratory interaction analysis among all the independent SNPs on the chip meeting quality filter criteria, irrespective of whether they demonstrated any individual effect. This analysis was performed to identify epistasis between variants that exhibit no marginal effects individually.

## Methods

### Study population

Discovery cohort

The discovery cohort consisted of 2,101 unrelated subjects with CAD originally recruited into the British Heart Foundation Family Heart Study (BHF-FHS) and 2,426 unrelated controls recruited into the Wellcome Trust Case Control Consortium (WTCCC). CAD was defined as history of myocardial infarction (MI), percutaneous coronary intervention or coronary artery bypass surgery prior to 66^th^ birthday, as reported before [14]. The control subjects were either recruited from the British 1958 Birth Cohort or from healthy blood donors as part of the common controls for the WTCCC genome-wide association (GWAS) study [15]. The phenotypic information available from the controls was limited to age, sex and geographic region of recruitment. Extensive analysis of the WTCCC GWAS data had shown no evidence of population stratification between cases and controls or between the two control groups [15].

Replication cohort

2,967 patients with early onset MI (≤ 50 years in men and ≤ 60 years in women) and 3,075 age and sex matched controls from Myocardial Infarction Genetics Consortium (MIGen) [16] was used as the replication sample. The database of Genotypes and Phenotypes (dbGaP; http://www.ncbi.nlm.nih.gov/gap; project number #2120) was the source for information on genotype and phenotype data used for the validation analysis.

### Ethics Statement

The original ethics approval was for the BHF-FHS study by the Northern and Yorkshire Research Ethics Committee. The approval number is MREC/0/3/2. The analysis that we did for this study was an in silico analysis on the original data which is covered by the original ethics and does not normally require a separate ethics. Written informed consent for inclusion was obtained previously from all the participants of both the discovery and replication studies [15,16].

### DNA analysis

In the discovery cohort, we used genotypes from IBC 50K array (version 1) with 45,707 single nucleotide polymorphisms (SNPs) spanning around 2,100 loci with high relevance to cardiovascular system, inflammation, lipids, diabetes and thrombosis [17,18], typed in both cases and controls. The selection of genes and loci typed on this array comprised a three tier system. Tier 1 included genes and loci known to contribute to cardiovascular disease as well as variants identified through GWAS at the time the array was developed in 2008 [17]. Loci that are potentially related to cardiovascular disease or those requiring high number of tags due to large locus size were included under Tier 2. Tier 3 included lower priority loci and also included non-synonymous and established functional SNPs. Quality control checks of genotyping of the discovery cohort did not show any batch effects [18].

The replication cohort was typed using the Affymetrix 6.0 GeneChip GWAS array and comprised 727,496 directly genotyped SNPs that passed quality filters as described previously [16].

### Statistical Analysis

SNP selection

Out of the 45,707 SNPs on the IBC array, 1,781 SNPs which belonged to ancestry informative and admixture controls were removed as were 39 SNPs that represented copy number variants and 600 SNPs located on the sex chromosomes. 12,018 rare SNPs (MAF < 1%), 8,487 low frequency SNP (MAF < 10%) and 759 SNPs with low (<90%) genotyping call rate were also removed. Application of a Hardy-Weinberg equilibrium P value cut-off of 1x10^-4^ in the controls led to the removal of 124 SNPs, leaving 21,899 SNPs.

**Fig 1:**
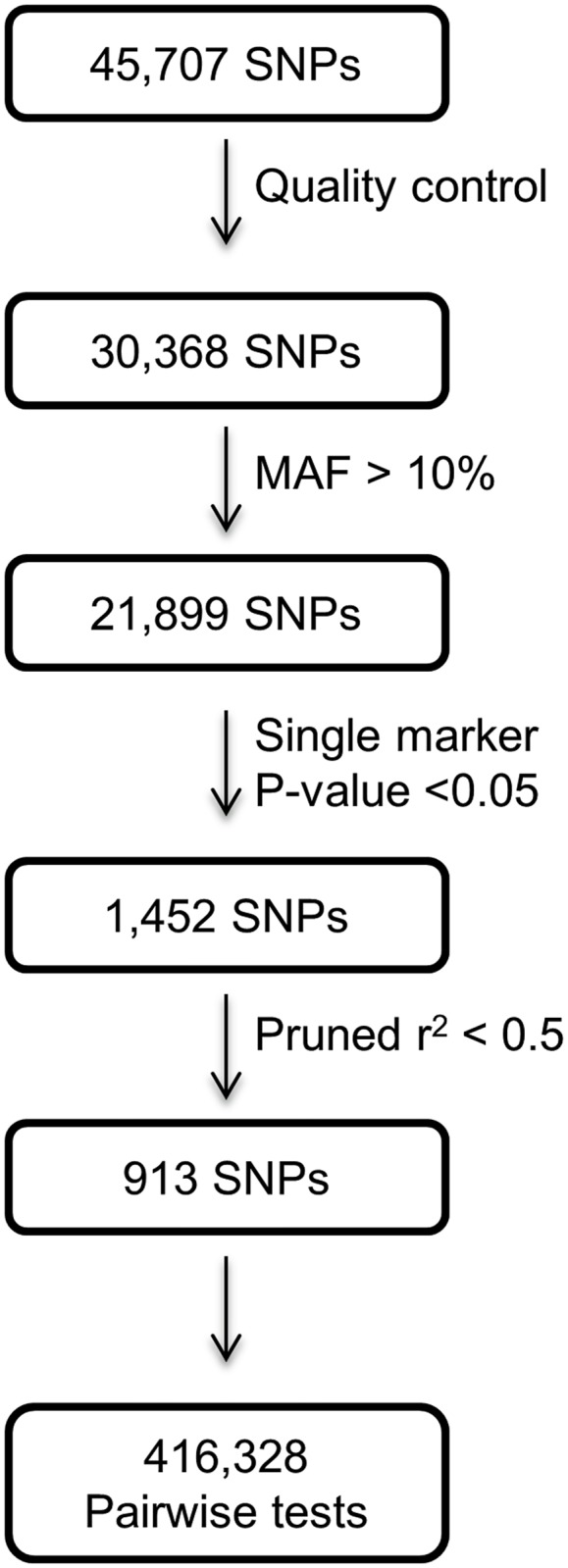
A schematic illustration of SNP selection process for the primary analysis.

**Fig 2:**
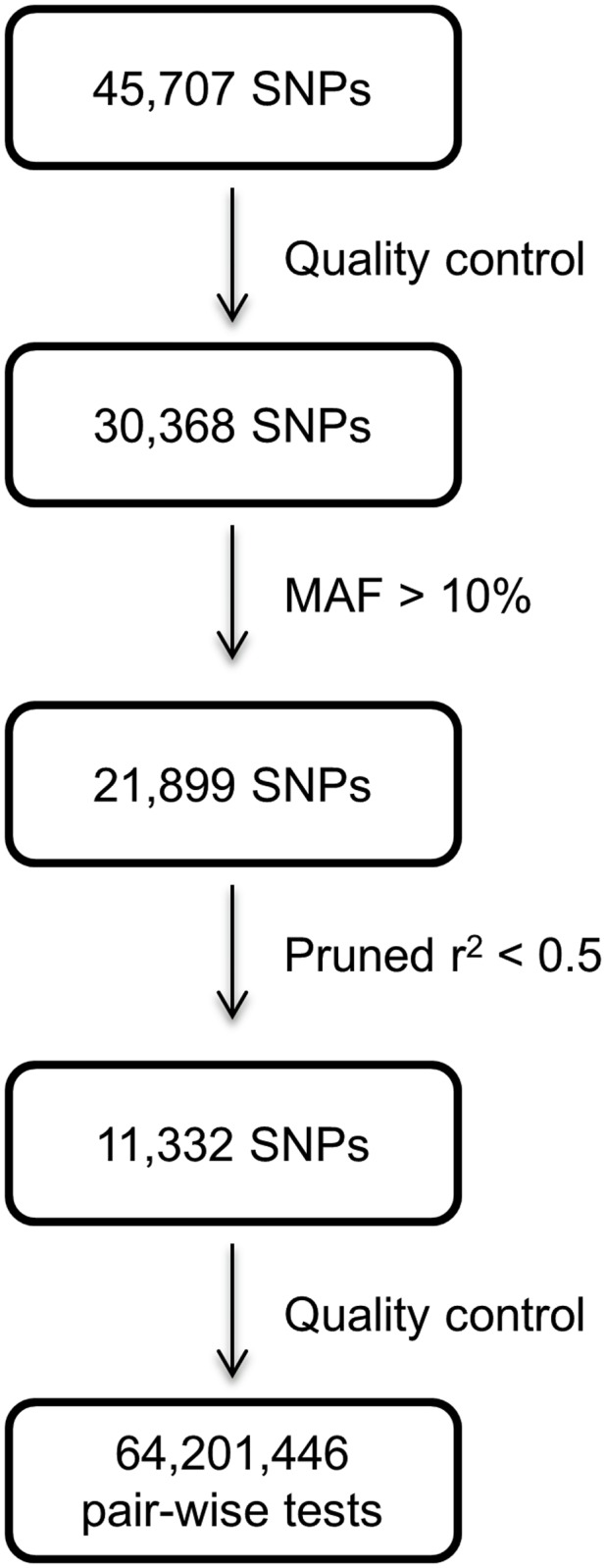
SNP selection process for the secondary analysis.

For the primary analysis, we identified SNPs that individually showed a nominal statistical association (P <0.05) with CAD, unadjusted, under an additive model analysed using logistic regression in PLINK as described before [18]. Of 1452 SNPs that met this criterion, pruning for independence (r^2^<0.5) left 913 independent SNPs that were included in the interaction analysis (Fig. 1). For the secondary analysis, of the 21,899 SNPs surviving quality filters, we took forward 11,332 SNPs that resulted from pruning for independence at r^2^ <0.5. These SNPs were included irrespective of their individual level of association with CAD (Fig. 2).

Interaction analysis

In all analyses we used a logistic regression test for additive allelic interaction adjusted for age and sex implemented in a freely available software package designed to examine genetic interactions—INTERSNP [10]. Assuming three SNPs, the allelic effect for each SNP i, i = 1,2,3 was modelled as x_i_ = -1,0,1 by coding the genotypes as (1,1), (1,2) and (2,2), respectively [10]. An interaction term was then derived by multiplying the individual allelic effect of each SNP. For example, the allelic interaction between SNPs 1 and 2 is denoted x_1_x_2_. *β*_*0*_ represents the baseline likelihood *L*_*0*_ = *logit(p)* = *β*_*0*_. The likelihood containing allelic terms only is denoted $L\begin{array}{c} A\\ 1,2 \end{array}$ = *β*_*0* +_ *β*_*1*_ *x*_*1* +_ *β*_*2*_ *x*_*2*_ whereas the likelihood containing both allelic and interaction terms is designated $L\begin{array}{c} A,I\\ 1,2 \end{array}$ = *β*_*0* +_ *β*_*1*_ *x*_*1* +_ *β*_*2*_ *x*_*2* +_ *β*_*1*,*2*_ *x*_*1*_ *x*_*2*_. The test we employed in this study (Test 5 in INTERSNP) compares $L\begin{array}{c} A\\ 1,2 \end{array}$ versus $L\begin{array}{c} A,I\\ 1,2 \end{array}$. This test has one degree of freedom.

For the secondary analysis, because of the size of the dataset we first used the logistic regression test for allelic interaction employed in PLINK (*—epistasis*). Adjustment for age or sex was not possible as this is not available in PLINK-based interaction commands. 579 interactions with at least suggestive significance (P<1.0x10^-5^) found in this analysis were then taken into INTERSNP to carry out age and sex adjusted pairwise allelic interaction analysis as described above.

Similar age and gender adjusted logistic regression analysis for interactions was undertaken in the replication cohort for the selected pairs of SNPs from the analysis of the discovery cohort.

Power calculation

Power to detect significant SNP-SNP interaction in the discovery cohort was calculated using Quanto programme [19]. The calculation was conducted assuming additive model for allelic interaction, MAF for both interacting alleles from 0.1 to 0.5, and a range of expected effect size of the interaction (odds ratio between 1.1 and 2.5). The power calculation was based on the primary analysis SNP sample (913 SNPs) and the resultant 416,328 independent interaction tests. Due to the large burden of multiple testing, the study had sufficient (>80%) power to detect only moderately large size effects (>1.7) of the interaction between the more common alleles (MAF > 0.2) when using Bonferroni correction. Full details of the power calculation are shown in the Table A in S1 File.

## Results

A summary of the baseline characteristics of both the discovery and replication cohorts is shown in Table 1.

**Table 1:** Baseline Characteristics of cases in the British Heart Foundation-Family Heart Study and the Myocardial Infarction Genetics studies.

N (%)	BHF-FHS	MIGen
n	2101	2943
Age (years)	60.1 (8.1)	42.82 (7.25)
Age at diagnosis (years)	49.8 (7.7)	42.82 (7.25)
Male (n, %)	1655 (78.8)	2287 (77.71%)
MI (n, %)	1538 (73.2)	2943 (100%)
Type 2 diabetes (n, %)	235 (11.2)	315 (10.70%)
Hypertension (n, %)	920 (43.8)	988 (33.57%)
Family history (n, %)	2101 (100)	-
BMI (kg/m^2^)	27.7 (4.3)	27.67 (5.14)
Current smoker (n, %)	914 (43.5)	1441 (48.96)

Data are means and standard deviations or counts and percentages, BHF-FHS: British Heart Foundation Family Heart Study; MIGen: Myocardial Infarction Genetics Consortium; MI: myocardial infarction; BMI: body mass index.

### Primary analysis

We tested for all possible pairwise interactions among the mutually independent 913 SNPs with at least nominal association with CAD in the single locus analysis. Out of the 416,328 interaction tests executed, none reached Bonferroni adjusted threshold for significance (P = 1.2x10^-7^). There were 6 SNP pairs with interaction at suggestive level of statistical significance (P<1 x10^-5^). The top interaction was identified between rs12989423 in phosphodiesterase11A (PDE11A) gene on chromosome 2 and rs8103121 in a pseudogene on chromosome 19 called secretory blood group 1, pseudogene (SEC1P) (P = 1.79x10^-6^) (Table 2). Of 49 pairs of SNPs that achieved interaction at P<1x10^-4^, a total of 36 SNP pairs could be evaluated in the replication cohort. None showed a statistically significant association with CAD after applying Bonferroni correction for multiple testing (1.4x10^-3^) (Table B in S1 File). There were no systematic differences in the allele frequencies of the SNP pairs taken for replication between the discovery and replication cohorts to explain the lack of replication based on differences in population sub-structure. The 913 SNPs used in this analysis included 9 SNPs that either was previously associated with CAD or tagging a SNP (r^2^ > 0.8) that was associated with CAD in GWAS reported by Deloukas et al [2]. None of these SNPs showed any significant interactions.

**Table 2:** Suggestive SNP-SNP interactions—primary analysis.

SNPA	ChrA	Gene	MAF	SNPA Pvalue	SNPB	ChrB	Gene	MAF	SNPB Pvalue	Int. Pvalue BHF-FHS	Int. Pvalue MIGen
rs12989423	2	PDE11A	0.10	9.90x10^-3^	rs8103121	19	SEC1P	0.34	2.67x10^-2^	1.79x10^-6^	4.05x10^-1^
rs17094917	14	SERPINA12	0.26	5.50x10^-3^	rs17150369	7	SRI	0.18	2.04x10^-2^	2.11x10^-6^	3.88x10^-3^
rs10095188	8	ZHX2	0.18	6.10x10^-3^	rs3790840	1	NR5A2	0.15	4.36x10^-2^	7.40x10^-6^	2.93x10^-1^
rs7248719	19	ANGPTL4	0.45	6.90x10^-3^	rs10884342	10	NRG3	0.28	3.02x10^-2^	7.67x10^-6^	NA
rs4464383	3	RPSAP15	0.33	1.70x10^-3^	rs10741762	11	CSRP3	0.16	2.70x10^-3^	8.57x10^-6^	NA
rs11101992	1	GSTM3	0.24	3.62x10^-2^	rs12131634	1	RYR2	0.15	3.68x10^-2^	9.97x10^-6^	NA
rs3779130	7	TBXAS1	0.34	3.20x10^-3^	rs4526299	7	TAC1	0.17	4.82x10^-2^	1.14x10^-5^	NA
rs25644	12	P2RX4	0.11	5.50x10^-3^	rs3816248	4	SCARB2	0.14	1.82x10^-2^	1.42x10^-5^	2.72x10^-1^
rs2709800	7	NOD1	0.43	5.60x10^-3^	rs901782	11	PDGFD	0.35	2.35x10^-2^	1.62x10^-5^	3.71x10^-1^

SNP: single nucleotide polymorphism, Chr: chromosome; MAF: minor allele frequency; BHF-FHS: British Heart Foundation Family Heart Study; MIGen: Myocardial Infarction Genetics Consortium; SNPA Pvalue: level of nominal statistical significance for single marker association with coronary artery disease for SNP A; SNPB Pvalue: level of nominal statistical significance for single marker association with coronary artery disease for SNP B; Int. Pvalue BHF-FHS: interaction P value in BHF-FHS; Int. Pvalue MIGen: interaction P value in MIGen; N/A: replication not available. PDE11A: phosphodiesterase 11A; SEC1P: secretory blood group 1, pseudogene; SERPINA12: serpin peptidase inhibitor, clade A; SRI: sorcin; ZHX2: zinc fingers and homeoboxes 2; NR5A2: nuclear receptor subfamily 5, group A, member 2; ANGPTL4: angiopoietin-like 4; NRG3: neuregulin 3; RPSAP15: ribosomal protein SA pseudogene 15; CSRP3: cysteine and glycine-rich protein 3; GSTM3: glutathione S-transferase mu 3; RYR2: ryanodine receptor 2; TBXAS1: thromboxane A synthase 1; TAC1: tachykinin, precursor 1; P2RX4: purinergic receptor P2X, ligand-gated ion channel, 4; SCARB2: scavenger receptor class B, member 2; NOD1: nucleotide-binding oligomerization domain containing 1; PDGFD: platelet derived growth factor D.

### Secondary analysis

The complete two locus analysis of 11,332 SNPs resulted in 64,204,446 tests. After applying Bonferroni cut-off for significance (calculated at P = 7.8x10^-10^), no statistically significant interactions were identified in this analysis. A total of 7 SNP pairs showed suggestive interaction (P<10^-6^) (Table 3). Of these the most significant was between rs9840469 in FERM domain containing 4B (FRMD4B) gene on chromosome 3 and rs10911935 in phospholipase A2, group IVA (PLA2G4A) gene on chromosome 1 (P = 4.6x10^-7^). After applying an arbitrary interaction threshold of suggestive significance (P = 1.0x10^-5^) 51 pairs of SNPs were taken forward for replication in the MIGen cohort. None of 33 pairs that could be evaluated for in the replication cohort exceeded the significance threshold based on Bonferroni correction (1.5x10^-3^) (Table C in S1 File).

**Table 3:** Suggestive SNP-SNP interactions—secondary analysis.

SNPA	ChrA	Gene	MAF	SNPA Pvalue	SNPB	ChrB	Gene	MAF	SNPB Pvalue	Int. Pvalue BHF-FHS	Int. PvalueMIGen
rs9840469	3	FRMD4B	0.38	7.00x10^-4^	rs10911935	1	PLA2G4A	0.21	4.84x10^-1^	4.63x10^-7^	NA
rs3759929	15	FURIN	0.39	7.29x10^-1^	rs780825	10	CUBN	0.31	9.54x10^-1^	5.18x10^-7^	NA
rs2740502	19	KLK1	0.40	6.61x10^-1^	rs7910038	10	PRKCQ	0.47	8.08x10^-1^	5.64x10^-7^	6.24x10^-1^
rs3748107	7	SBDS	0.28	2.95x10^-1^	rs7130671	11	NCAM1	0.35	3.48x10^-1^	6.2x10^-7^	7.73x10^-2^
rs7629902	3	RARB	0.15	2.03x10^-1^	rs6472228	8	PDE7A	0.15	5.59x10^-1^	7.26x10^-7^	8.98x10^-1^
rs2245121	10	SFTPD	0.43	2.28x10^-1^	rs4418583	1	LDLRAP1	0.50	7.18x10^-1^	7.51x10^-7^	NA
rs613089	1	BCL9	0.31	6.00x10^-1^	rs11466521	3	TGFBR2	0.23	9.28x10^-1^	8.79x10^-7^	NA
rs9862	22	TIMP3	0.49	3.40x10^-1^	rs753424	1	HMGCS2	0.46	5.34x10^-1^	1.09x10^-6^	3.01x10^-1^
rs11807878	1	BCL9	0.12	1.14x10^-1^	rs12060491	1	PDE4B	0.17	1.82x10^-1^	1.15x10^-6^	1.61x10^-2^

SNP: single nucleotide polymorphism, Chr: chromosome; MAF: minor allele frequency; BHF-FHS: British Heart Foundation Family Heart Study; MIGen: Myocardial Infarction Genetics Consortium; SNPA Pvalue: level of nominal statistical significance for single marker association with coronary artery disease for SNP A; SNPB Pvalue: level of nominal statistical significance for single marker association with coronary artery disease for SNP B; Int. Pvalue BHF-FHS: interaction P value in BHF-FHS; Int. Pvalue MIGen: interaction P value in MIGen; N/A: replication not available. FRMD4B: FERM domain containing 4B; PLA2G4A: phospholipase A2, group IVA; FURIN: furin (paired basic amino acid cleaving enzyme); CUBN: cubilin (intrinsic factor-cobalamin receptor); KLK1: kallikrein 1; PRKCQ protein kinase C, theta; SBDS: Shwachman-Bodian-Diamond syndrome; NCAM1: neural cell adhesion molecule 1; RARB: retinoic acid receptor, beta; PDE7A: phosphodiesterase 7A; SFTPD: surfactant protein D; LDLRAP1: low density lipoprotein receptor adaptor protein 1; BCL9: B-cell CLL/lymphoma 9; TGFBR2: transforming growth factor, beta receptor II (70/80kDa); TIMP3: TIMP metallopeptidase inhibitor 3; HMGCS2: 3-hydroxy-3-methylglutaryl-CoA synthase 2 (mitochondrial); PDE4B: phosphodiesterase 4B, cAMP-specific.

## Discussion

Here we report findings from a systematic evaluation of gene-gene interactions in coronary artery disease. Using a selective policy focusing on common variants in cardiovascular genes, we show that none of the analysed interactions survived correction for multiple testing and the majority of the suggestive interactions were not replicated in the validation cohort. These findings indicate a lack of moderately large size additive gene-gene interactions amongst common genetic variants based on known cardiovascular pathways.

The major proportion of heritability of CAD remains unexplained despite the flurry of data produced by GWAS. The ~50 loci identified to date through large international collaborations only account for ~10% of CAD heritability [2]. The remainder of the heritability may be attributed to other factors such as rare variants with large effects, gene-environment interactions, gene-gene interactions and epigenetic factors [20]. The evidence emerging from both experimental studies and association analyses suggests that gene-gene interactions may contribute to inter-individual variation in susceptibility to complex diseases [6,21–24]. Several studies have attempted to delineate gene-gene interactions in relation to a variety of complex traits with very limited success [3]. A number of practical challenges plagued these endeavours to uncover gene-gene interactions including the heavy penalty for multiple testing which renders standard GWAS samples underpowered to detect such effects. Furthermore, such exhaustive analyses can be computationally prohibitive and require massive parallelisation [10]. Various strategies have been developed to improve the power to identify gene-gene interactions in GWAS datasets [3]. For example, prioritising SNPs for interaction analysis based on their performance in the single-marker GWAS analysis [9,12]. Incorporating knowledge from protein-protein interactions and biological pathways are other potentially appropriate criteria for candidate SNP selection [25–27]. While gene-gene interactions are more likely to take place between loci that show some effects individually, pure epistatic loci may contribute to the genetic risk variation without having single locus effects. Such loci can be overlooked if not tested for specifically [8].

In this study we minimised our search space for gene-gene interactions by selecting the IBC 50K chip which is enriched for genetic markers in or near genes linked to the cardiovascular system and related risk factors. This reduced the starting number of SNPs from over 500,000 in a standard GWAS array to just under 50,000 SNPs. Using biological pathway information to prioritise genetic loci for interaction analysis proved successful in certain complex traits and conditions such as asthma [28,29] and lipid levels [5]. By virtue of its design, the IBC 50K array provides good coverage for loci related to cardiovascular, inflammatory and metabolic syndromes and hence provides indirect strategy for utilising pathway knowledge [17]. It is however important to note that the IBC 50K array was designed a few years ago, and therefore many of the CAD loci discovered in the past three years are not included on the array.

The statistical approach we employed here is based on logistic regression models since regression models are thought to be one of the most efficient ways to investigate interacting genetic loci that modify disease risk [3]. We have also elected to use an allelic test for interaction (1 degree of freedom) as opposed to a genotype test for interaction (4 degrees of freedom) because the latter has more degrees of freedom and hence will require larger sample size to have equivalent power. Mindful of the limited sample size available for this study we sought to examine potential interactions among the more common variants with MAF >10% as this allowed enough power to detect moderate interaction effects (OR > 1.7).

While our primary analysis was meant to maximise the chances to detect gene-gene interactions within a relatively small sample of candidate SNPs with at least nominal association with CAD, our secondary exploratory analysis was implemented to ensure that potential pure epistatic SNPs that have no marginal effects on their own are captured by this study. Recognising the very limited power of this analysis we did not find any replicated interactions under this model either.

Our primary analytical approach is similar to that recently employed by Lucas et al. [9] who tested a biological hypothesis, under which MI risk is modulated by interactions between variants that are known to be relevant for its risk factors; and a statistical hypothesis, under which interacting variants individually show weak marginal association with MI were examined. Consistent with our findings they did not find any significant interactions. Compared to the work by Lucas et al, our analysis has the potential advantage of not being restricted to those variants which were previously significantly associated with cardiovascular risk factors. Additionally we were able to implement a large scale search for gene-gene interactions among cardiovascular genes benefiting from the IBC 50K array that was not feasible on the GWAS platform used by Lucas et al [9]. Other groups have used different methods to examine gene-gene interactions bearing in mind the methodical and computational challenges. For example, Wan et al [30] were able to examine pairwise interactions efficiently in GWAS data using a method called Boolean Operation-based Screening and Testing (BOOST). They claim to have achieved increased testing efficiency through fast logic operations made possible by the Boolean representation of genotype data. They did not identify any significant interactions in relation to CAD which is consistent with our findings. Recently, Lippert et al demonstrated that one can increase the power of interaction studies through combining data sets [31] and using certain disease cohorts as controls for other diseases. They exploited linear mixed models to overcome relatedness and population structure. Using genotypes available from the Wellcome Trust Case Control Consortium (WTCCC) GWAS study, they identified 42 SNP-SNP interactions related to the risk of CAD, although these findings require further validation.

Our study has several limitations. The principal one relates to low power arising from a combination of the modest size of the discovery cohort and the heavy penalty from the correction required for the large number of interactions analysed. To limit the impact of these issues we decided a priori to only analyse common variants. Despite this we only had sufficient power to detect relatively high odds ratios although in the range that one might have anticipated for powerful interactions, if these exist. Therefore our study does not exclude interactions between lower frequency variants or those that are of modest strength even within the candidate cardiovascular genes analysed. In addition, the true underlying interaction model may differ from the one we applied in this study. However, there is a trade-off between choosing a highly parameterized model such as the saturated model that is very likely to encompass the true underlying model at the expense of higher degrees of freedom and the resulting decreased power [3]. Applying novel strategies such as adaptive locus-based validation as demonstrated by Liu et al [32] and applied by Ma et al [5] also shows promise especially as more sequencing data is becoming available. Finally, we have only analysed a very small proportion of the totality of genomic variation, albeit in genes with biological plausibility.

In summary, we found no evidence of large effect interactions amongst common variants in a large set of cardiovascular system related genes impacting on CAD risk. The key challenge for gene-gene interactions moving forward is whether sufficiently large sample sizes with individual level data will become available to undertake genome-wide analysis and whether analytical approaches can be developed which can distinguish true interactions amongst the large number of possibilities examined.

## Supporting Information

S1 File Supplementary material containing Supplementary tables A-D. (DOCX)
