# Supplementary material for: Analysis of Gene-Gene Interactions among Common Variants in Candidate Cardiovascular Genes in Coronary Artery Disease
Source: PLoS One. 2015 Feb 6;10(2):e0117684. doi: 10.1371/journal.pone.0117684 (PMC4320092; doi:10.1371/journal.pone.0117684)
Supplement: S1 File — (DOCX) [file pone.0117684.s001.docx]

**Analysis of gene-gene interactions among common variants in coronary artery disease**

Muntaser D Musameh^1,2^, William Y S Wang^3^, Christopher P Nelson^1,2^, [Carla Lluís-Ganella](http://www.ncbi.nlm.nih.gov/pubmed/?term=Llu%26%23x000ed%3Bs-Ganella%20C%5Bauth%5D)^4^, [Radoslaw Debiec](http://www.ncbi.nlm.nih.gov/pubmed/?term=Debiec%20R%5Bauth%5D)^1,2^, Isaac Subirana^4,5^, Roberto Elosua^4^, Anthony J. Balmforth^7^**,** Stephen G Ball^6^, Alistair S Hall^7^, [Sekar Kathiresan](http://www.ncbi.nlm.nih.gov/pubmed/?term=Kathiresan%20S%5Bauth%5D)^8^, John R Thompson^1^, [[Gavin Lucas](http://www.ncbi.nlm.nih.gov/pubmed/?term=Lucas%20G%5Bauth%5D)^4^](http://www.ncbi.nlm.nih.gov/pubmed/?term=Elosua%20R%5Bauth%5D) , Nilesh J Samani^1,2^, Maciej Tomaszewski^1,2^

^1^ Department of Cardiovascular Sciences, University of Leicester, Glenfield Hospital, Leicester, UK

^2^ Leicester NIHR Biomedical Research Unit in Cardiovascular Disease, Glenfield Hospital, Leicester, UK

^3^ School of Medicine, University of Queensland, Brisbane, Queensland, Australia

^4^ Cardiovascular Epidemiology and Genetics, IMIM, Barcelona, Spain

^5^ Epidemiology and Public Health Network (CIBERESP), Barcelona, Spain

^6^ University of Leeds, MCRC, Leeds Institute of Genetics, Health and Therapeutics, Leeds, United Kingdom

^7^ Division of Epidemiology, LIGHT, School of Medicine, University of Leeds, Leeds, UK

^8^ The Broad Institute of MIT and Harvard, Cambridge, Massachusetts, United States of America

Correspondence to:

Dr Muntaser D Musameh, Department of Cardiovascular Sciences, University of Leicester, Glenfield Hospital, Leicester, LE3 9QP, Leicester, UK

Tel: +44 116 2044761; Fax: +44 116 2875792; email: mdmm2@le.ac.uk

**Supplementary Table A. Power calculation – discovery cohort (British Heart Foundation-Family Heart Study/Wellcome Trust Case Control Consortium)**

| **Allele 1 MAF** | **0.1** | | | | | **0.2** | | | | **0.3** | | | **0.4** | | | **0.5** | |
| --- | --- | --- | --- | --- | --- | --- | --- | --- | --- | --- | --- | --- | --- | --- | --- | --- | --- |
| **Allele 2 MAF** | **0.1** | **0.2** | **0.3** | **0.4** | **0.5** | **0.2** | **0.3** | **0.4** | **0.5** | **0.3** | **0.4** | **0.5** | **0.4** | **0.5** | **0.5** | |  |
| **OR** | **POWER** | | | | | | | | | | | | | | | | |
| **1.1** | 0.00 | 0.00 | 0.00 | 0.00 | 0.00 | 0.00 | 0.00 | 0.00 | 0.00 | 0.00 | 0.01 | 0.01 | 0.01 | 0.01 | 0.01 | |  |
| **1.2** | 0.00 | 0.01 | 0.02 | 0.02 | 0.02 | 0.04 | 0.11 | 0.18 | 0.20 | 0.31 | 0.51 | 0.58 | 0.85 | 0.99 | 1.16 | |  |
| **1.3** | 0.01 | 0.08 | 0.21 | 0.33 | 0.36 | 0.72 | 1.98 | 3.14 | 3.45 | 5.44 | 8.56 | 9.53 | 13.42 | 15.12 | 17.30 | |  |
| **1.4** | 0.07 | 0.60 | 1.56 | 2.37 | 2.49 | 5.23 | 12.94 | 18.78 | 20.01 | 29.10 | 39.95 | 42.65 | 53.15 | 56.83 | 61.27 | |  |
| **1.5** | 0.33 | 2.73 | 6.74 | 9.66 | 9.92 | 19.82 | 39.74 | 50.90 | 52.61 | 66.78 | 78.34 | 80.48 | 88.17 | 90.18 | 92.37 | |  |
| **1.6** | 1.12 | 8.67 | 19.02 | 25.34 | 25.51 | 45.31 | 71.10 | 80.87 | 81.87 | 91.27 | 96.05 | 96.67 | 98.67 | 99.03 | 99.38 | |  |
| **1.7** | 3.08 | 20.31 | 38.42 | 47.35 | 47.06 | 71.66 | 90.76 | 95.27 | 95.55 | 98.70 | 99.62 | 99.70 | 99.93 | 99.96 | 99.98 | |  |
| **1.8** | 7.04 | 37.23 | 60.13 | 68.98 | 68.21 | 89.03 | 98.00 | 99.23 | 99.27 | 99.89 | 99.98 | 99.98 | 99.99 | 99.99 | 99.99 | |  |
| **1.9** | 13.73 | 56.15 | 78.20 | 84.73 | 83.82 | 96.79 | 99.70 | 99.91 | 99.92 | 99.99 | 99.99 | 99.99 | 99.99 | 99.99 | 99.99 | |  |
| **2.0** | 23.33 | 73.03 | 89.88 | 93.64 | 92.93 | 99.27 | 99.97 | 99.99 | 99.99 | 99.99 | 99.99 | 99.99 | 99.99 | 99.99 | 99.99 | |  |
| **2.1** | 35.27 | 85.37 | 95.97 | 97.72 | 97.29 | 99.87 | 99.99 | 99.99 | 99.99 | 99.99 | 99.99 | 99.99 | 99.99 | 99.99 | 99.99 | |  |
| **2.2** | 48.36 | 92.96 | 98.60 | 99.28 | 99.08 | 99.98 | 99.99 | 99.99 | 99.99 | 99.99 | 99.99 | 99.99 | 99.99 | 99.99 | 99.99 | |  |
| **2.3** | 61.16 | 96.97 | 99.57 | 99.80 | 99.71 | 99.99 | 99.99 | 99.99 | 99.99 | 99.99 | 99.99 | 99.99 | 99.99 | 99.99 | 99.99 | |  |
| **2.4** | 72.46 | 98.82 | 99.88 | 99.95 | 99.92 | 99.99 | 99.99 | 99.99 | 99.99 | 99.99 | 99.99 | 99.99 | 99.99 | 99.99 | 99.99 | |  |
| **2.5** | 81.57 | 99.58 | 99.97 | 99.99 | 99.98 | 99.99 | 99.99 | 99.99 | 99.99 | 99.99 | 99.99 | 99.99 | 99.99 | 99.99 | 99.99 | |  |

MAF: minor allele frequency; OR: odds ratios, the shaded area represents power ≥ 80 % for detecting interactions at different allele frequencies and interaction effect sizes.

### Supplementary Table B. Top SNP-SNP interactions - primary analysis

| **SNPA** | **ChrA** | **SNPA Pvalue** | **SNP B** | **ChrB** | **SNPB Pvalue** | **Int. Pvalue BHF-FHS** | **Int. Pvalue MIGen** |
| --- | --- | --- | --- | --- | --- | --- | --- |
| rs12989423 | 2 | 9.91x10^-3^ | rs8103121 | 19 | 2.67x10^-2^ | 1.79x10^-6^ | 4.05x10^-1^ |
| rs17094917 | 14 | 5.52x10^-3^ | rs17150369 | 7 | 2.04x10^-2^ | 2.11x10^-6^ | 3.88x10^-3^ |
| rs10095188 | 8 | 6.11x10^-3^ | rs3790840 | 1 | 4.36x10^-2^ | 7.40x10^-6^ | 2.93x10^-1^ |
| rs7248719 | 19 | 6.88x10^-3^ | rs10884342 | 10 | 3.02x10^-2^ | 7.67x10^-6^ | NA |
| rs4464383 | 3 | 1.67x10^-3^ | rs10741762 | 11 | 2.69x10^-3^ | 8.57x10^-6^ | NA |
| rs11101992 | 1 | 3.62x10^-2^ | rs12131634 | 1 | 3.68x10^-2^ | 9.97x10^-6^ | NA |
| rs3779130 | 7 | 3.22x10^-3^ | rs4526299 | 7 | 4.82x10^-2^ | 1.14x10^-5^ | NA |
| rs25644 | 12 | 5.48x10^-3^ | rs3816248 | 4 | 1.82x10^-2^ | 1.42x10^-5^ | 2.72x10^-1^ |
| rs2709800 | 7 | 5.58x10^-3^ | rs901782 | 11 | 2.35x10^-2^ | 1.62x10^-5^ | 3.71x10^-1^ |
| rs4616271 | 15 | 5.46x10^-3^ | rs6684865 | 1 | 8.74x10^-3^ | 1.90x10^-5^ | 2.82x10^-1^ |
| rs7933420 | 11 | 3.34x10^-3^ | rs10787010 | 10 | 3.95x10^-2^ | 1.95x10^-5^ | NA |
| rs783144 | 6 | 6.88x10^-3^ | rs2000999 | 16 | 1.17x10^-2^ | 2.35x10^-5^ | 8.86x10^-1^ |
| rs12904906 | 15 | 7.21x10^-3^ | rs17269264 | 15 | 3.88x10^-2^ | 2.53x10^-5^ | 7.53x10^-1^ |
| rs17334072 | 2 | 3.47x10^-2^ | rs12640088 | 4 | 3.78x10^-2^ | 2.63x10^-5^ | 1.97x10^-1^ |
| rs994901 | 12 | 1.95x10^-2^ | rs2110902 | 6 | 2.44x10^-2^ | 2.72x10^-5^ | 7.47x10^-1^ |
| rs1653618 | 12 | 1.87x10^-2^ | rs6790343 | 3 | 5.24x10^-2^ | 2.85x10^-5^ | 9.09x10^-1^ |
| rs10889923 | 1 | 4.57x10^-2^ | rs783176 | 6 | 4.70x10^-2^ | 2.86x10^-5^ | 6.14x10^-1^ |
| rs1722845 | 3 | 4.36x10^-2^ | rs1341023 | 20 | 4.43x10^-2^ | 3.42x10^-5^ | 6.19x10^-1^ |
| rs2684799 | 15 | 1.41x10^-2^ | rs7111879 | 11 | 3.75x10^-2^ | 3.65x10^-5^ | 9.20x10^-1^ |
| rs17720953 | 18 | 7.65x10^-3^ | rs10925478 | 1 | 2.59x10^-2^ | 3.69x10^-5^ | 9.99x10^-1^ |
| rs3753753 | 1 | 1.52x10^-2^ | rs1380 | 9 | 3.21x10^-2^ | 3.82x10^-5^ | 7.78x10^-1^ |
| rs924191 | 12 | 1.87x10^-2^ | rs9607850 | 22 | 3.49x10^-2^ | 3.88x10^-5^ | NA |
| rs7250581 | 19 | 2.92x10^-4^ | rs4977982 | 9 | 9.57x10^-3^ | 4.08x10^-5^ | 4.55x10^-1^ |
| rs11207997 | 1 | 1.83x10^-2^ | rs479504 | 3 | 3.46x10^-2^ | 4.53x10^-5^ | 5.26x10^-2^ |
| rs9858542 | 3 | 1.68x10^-2^ | rs1485766 | 4 | 3.50x10^-2^ | 4.73x10^-5^ | NA |
| rs1277306 | 4 | 1.56x10^-2^ | rs10047420 | 11 | 2.58x10^-2^ | 4.80x10^-5^ | 1.57x10^-1^ |
| rs17747257 | 4 | 1.28x10^-2^ | rs1205399 | 2 | 1.35x10^-2^ | 4.93x10^-5^ | 9.92x10^-1^ |
| rs2302074 | 4 | 9.89x10^-3^ | rs11611246 | 12 | 2.64x10^-2^ | 4.97x10^-5^ | 3.85x10^-1^ |
| rs2180696 | 20 | 1.04x10^-3^ | rs752637 | 7 | 4.52x10^-2^ | 5.04x10^-5^ | NA |
| rs2547438 | 19 | 2.86x10^-3^ | rs476496 | 20 | 1.44x10^-2^ | 5.06x10^-5^ | NA |
| rs4246650 | 2 | 2.55x10^-2^ | rs4512969 | 13 | 3.17x10^-2^ | 5.51x10^-5^ | NA |
| rs6798507 | 3 | 3.82x10^-2^ | rs4894030 | 2 | 4.60x10^-2^ | 5.89x10^-5^ | 8.70x10^-1^ |
| rs743562 | 5 | 9.44x10^-3^ | rs758185 | 17 | 2.00x10^-2^ | 5.94x10^-5^ | 6.81x10^-1^ |
| rs1288344 | 1 | 1.26x10^-2^ | rs1692804 | 8 | 1.74x10^-2^ | 6.28x10^-5^ | 7.20x10^-1^ |
| rs4508523 | 19 | 9.41x10^-3^ | rs7611476 | 3 | 1.99x10^-2^ | 6.67x10^-5^ | 3.38x10^-1^ |
| rs17334072 | 2 | 3.47x10^-2^ | rs7938751 | 11 | 5.13x10^-2^ | 6.71x10^-5^ | 5.63x10^-1^ |
| rs9717605 | 6 | 7.27x10^-3^ | rs11574026 | 12 | 4.53x10^-2^ | 6.73x10^-5^ | 1.70x10^-1^ |
| rs5065 | 1 | 1.13x10^-2^ | rs17574203 | 1 | 4.62x10^-2^ | 6.73x10^-5^ | 2.99x10^-1^ |
| rs2312586 | 19 | 1.18x10^-2^ | rs235711 | 20 | 2.83x10^-2^ | 7.10x10^-5^ | NA |
| rs718567 | 12 | 1.82x10^-2^ | rs5030095 | 3 | 2.56x10^-2^ | 7.24x10^-5^ | 5.77x10^-1^ |
| rs10431700 | 14 | 4.96x10^-6^ | rs6443950 | 3 | 3.09x10^-2^ | 7.35x10^-5^ | 9.98x10^-1^ |
| rs4688982 | 4 | 1.95x10^-2^ | rs4973608 | 2 | 4.88x10^-2^ | 7.91x10^-5^ | 1.86x10^-1^ |
| rs2206577 | 6 | 4.26x10^-2^ | rs10889923 | 1 | 4.57x10^-2^ | 8.25x10^-5^ | 7.47x10^-2^ |
| rs241429 | 6 | 3.20x10^-3^ | rs4897554 | 6 | 2.12x10^-2^ | 8.48x10^-5^ | 8.70x10^-1^ |
| rs9840469 | 3 | 6.94x10^-4^ | rs7045889 | 9 | 1.04x10^-2^ | 8.62x10^-5^ | 5.95x10^-1^ |
| rs13283456 | 9 | 3.00x10^-2^ | rs12374408 | 4 | 4.28x10^-2^ | 8.89x10^-5^ | NA |
| rs1049434 | 1 | 3.18x10^-2^ | rs17377240 | 1 | 3.42x10^-2^ | 8.90x10^-5^ | 1.17x10^-1^ |
| rs6265 | 11 | 6.90x10^-4^ | rs8103121 | 19 | 2.67x10^-2^ | 9.11x10^-5^ | 7.57x10^-1^ |
| rs2820312 | 1 | 2.46x10^-3^ | rs4896219 | 6 | 7.25x10^-3^ | 9.67x10^-5^ | NA |

SNP: single nucleotide polymorphism; Chr: chromosome; SNPA Pvalue: level of nominal statistical significance for single marker association with coronary artery disease for SNP A; SNPB Pvalue: level of nominal statistical significance for single marker association with coronary artery disease for SNP B; BHF-FHS: British Heart Foundation Family Heart Study; MIGen: Myocardial Infarction Genetics Consortium; Int. Pvalue BHF-FHS: interaction P value in BHF-FHS; Int. Pvalue MIGen: interaction P value in MIGen; N/A: replication not available.

**Supplementary Table C. Top SNP-SNP interactions - secondary analysis**

| **SNPA** | **Chr A** | **SNPA**  **Pvalue** | **SNP B** | **ChrB** | **SNPB**  **Pvalue** | **Int. Pvalue**  **BHF-FHS** | **Int. Pvalue**  **MIGen** |
| --- | --- | --- | --- | --- | --- | --- | --- |
| rs9840469 | 3 | 7.08x10^-4^ | rs10911935 | 1 | 4.84x10^-1^ | 4.63x10^-7^ | NA |
| rs3759929 | 15 | 7.29x10^-1^ | rs780825 | 10 | 9.54x10^-1^ | 5.18x10^-7^ | NA |
| rs2740502 | 19 | 6.61x10^-1^ | rs7910038 | 10 | 8.08x10^-1^ | 5.64x10^-7^ | 6.24x10^-1^ |
| rs3748107 | 7 | 2.95x10^-1^ | rs7130671 | 11 | 3.48x10^-1^ | 6.20x10^-7^ | 7.73x10^-2^ |
| rs7629902 | 3 | 2.03x10^-1^ | rs6472228 | 8 | 5.59x10^-1^ | 7.26x10^-7^ | 8.98x10^-1^ |
| rs2245121 | 10 | 2.28x10^-1^ | rs4418583 | 1 | 7.18x10^-1^ | 7.51x10^-7^ | NA |
| rs613089 | 1 | 6.00x10^-1^ | rs11466521 | 3 | 9.28x10^-1^ | 8.79x10^-7^ | NA |
| rs9862 | 22 | 3.40x10^-1^ | rs753424 | 1 | 5.34x10^-1^ | 1.09x10^-6^ | 3.01x10^-1^ |
| rs11807878 | 1 | 1.14x10^-1^ | rs12060491 | 1 | 1.82x10^-1^ | 1.15x10^-6^ | 1.61x10^-2^ |
| rs3024317 | 6 | 3.76x10^-2^ | rs12703165 | 7 | 7.65x10^-1^ | 1.29x10^-6^ | NA |
| rs245082 | 5 | 9.20x10^-2^ | rs12046641 | 1 | 3.26x10^-1^ | 1.34x10^-6^ | NA |
| rs6413504 | 19 | 5.62x10^-2^ | rs6729914 | 2 | 3.91x10^-1^ | 1.50x10^-6^ | 4.24x10^-3^ |
| rs1454102 | 15 | 2.38x10^-1^ | rs17511192 | 1 | 3.30x10^-1^ | 1.57x10^-6^ | 1.86x10^-1^ |
| rs4872056 | 8 | 3.97x10^-1^ | rs892035 | 19 | 4.78x10^-1^ | 2.27x10^-6^ | 6.80x10^-1^ |
| rs17094917 | 14 | 5.76x10^-3^ | rs17150369 | 7 | 1.95x10^-2^ | 2.47x10^-6^ | 3.88x10^-3^ |
| rs6006427 | 22 | 5.81x10^-1^ | rs666004 | 11 | 6.67x10^-1^ | 2.72x10^-6^ | 5.30x10^-1^ |
| rs459546 | 11 | 2.00x10^-1^ | rs11602936 | 11 | 8.82x10^-1^ | 2.94x10^-6^ | 8.58x10^-1^ |
| rs6765267 | 3 | 6.46x10^-2^ | rs7217186 | 17 | 9.16x10^-2^ | 3.24x10^-6^ | 4.10x10^-1^ |
| rs6557421 | 6 | 4.91x10^-1^ | rs17654278 | 4 | 9.16x10^-1^ | 3.26x10^-6^ | 4.82x10^-1^ |
| rs8177352 | 11 | 3.42x10^-1^ | rs2238140 | 12 | 5.72x10^-1^ | 3.63x10^-6^ | NA |
| rs2267667 | 6 | 5.28x10^-1^ | rs983230 | 9 | 9.17x10^-1^ | 3.87x10^-6^ | 2.62x10^-1^ |
| rs770266 | 13 | 6.62x10^-1^ | rs12451302 | 17 | 9.37x10^-1^ | 4.22x10^-6^ | NA |
| rs11167496 | 5 | 7.07x10^-1^ | rs727139 | 3 | 9.43x10^-1^ | 4.31x10^-6^ | 9.81x10^-1^ |
| rs2453021 | 1 | 4.61x10^-1^ | rs4385869 | 11 | 4.76x10^-1^ | 4.43x10^-6^ | NA |
| rs2740434 | 8 | 6.41x10^-1^ | rs3917768 | 1 | 7.30x10^-1^ | 4.67x10^-6^ | 6.33x10^-1^ |
| rs12765044 | 10 | 9.24x10^-2^ | rs2885370 | 3 | 3.30x10^-1^ | 4.86x10^-6^ | NA |
| rs1466997 | 4 | 5.16x10^-1^ | rs12448206 | 16 | 6.82x10^-1^ | 5.06x10^-6^ | 5.30x10^-1^ |
| rs1808563 | 19 | 2.23x10^-1^ | rs12478449 | 2 | 4.41x10^-1^ | 5.17x10^-6^ | NA |
| rs4879923 | 9 | 1.67x10^-1^ | rs11134527 | 5 | 4.60x10^-1^ | 5.28x10^-6^ | 1.83x10^-2^ |
| rs10778306 | 12 | 3.90x10^-1^ | rs12485273 | 3 | 4.46x10^-1^ | 6.10x10^-6^ | 6.06x10^-1^ |
| rs4792147 | 17 | 5.31x10^-1^ | rs2425640 | 20 | 5.85x10^-1^ | 6.24x10^-06^ | 7.87x10^-1^ |
| rs4385869 | 11 | 4.76x10^-1^ | rs2493215 | 1 | 5.88x10^-1^ | 6.34x10^-6^ | NA |
| rs928670 | 10 | 6.74x10^-1^ | rs12439075 | 15 | 7.99x10^-1^ | 7.18x10^-6^ | 8.42x10^-1^ |
| rs3791575 | 2 | 2.70x10^-1^ | rs740076 | 5 | 7.22x10^-1^ | 7.27x10^-6^ | 8.90x10^-1^ |
| rs3136534 | 4 | 1.59x10^-1^ | rs1895604 | 12 | 9.17x10^-1^ | 7.39x10^-6^ | NA |
| rs3887446 | 17 | 3.77x10^-1^ | rs484208 | 6 | 8.78x10^-1^ | 7.42x10^-6^ | 2.26x10^-1^ |
| rs12566535 | 1 | 1.22x10^-1^ | rs12603094 | 17 | 8.19x10^-1^ | 7.74x10^-6^ | 5.52x10^-1^ |
| rs7757388 | 6 | 5.55x10^-1^ | rs2237882 | 11 | 6.93x10^-1^ | 7.85x10^-6^ | 3.08x10^-1^ |
| rs7821186 | 8 | 3.20x10^-1^ | rs1283816 | 12 | 3.98x10^-1^ | 8.10x10^-6^ | 2.63x10^-1^ |
| rs7983466 | 13 | 1.55x10^-1^ | rs4695148 | 4 | 6.68x10^-1^ | 8.56x10^-6^ | 4.80x10^-1^ |
| rs154556 | 5 | 7.38x10^-2^ | rs982076 | 4 | 1.70x10^-1^ | 8.66x10^-6^ | 7.49x10^-1^ |
| rs11740792 | 5 | 3.83x10^-1^ | rs10773111 | 12 | 9.82x10^-1^ | 8.74x10^-6^ | 1.62x10-^1^ |
| rs4810479 | 20 | 3.01x10^-1^ | rs9651118 | 1 | 9.18x10^-1^ | 8.90x10^-6^ | NA |
| rs11080994 | 18 | 1.53x10^-1^ | rs911122 | 20 | 2.37x10^-1^ | 8.97x10^-6^ | NA |
| rs12199523 | 6 | 7.89x10^-1^ | rs1233400 | 6 | 8.29x10^-1^ | 9.25x10^-6^ | 7.80x10^-1^ |
| rs7300068 | 12 | 6.89x10^-1^ | rs5517 | 19 | 9.85x10^-1^ | 9.27x10^-6^ | 5.28x10^-2^ |
| rs2076074 | 1 | 3.88x10^-1^ | rs2740434 | 8 | 6.41x10^-1^ | 9.64x10^-6^ | NA |
| rs4531272 | 1 | 6.61x10^-1^ | rs10925402 | 1 | 7.33x10^-1^ | 9.77x10^-6^ | 7.56x10^-1^ |
| rs12765044 | 10 | 9.24x10^-2^ | rs1988743 | 3 | 7.40x10^-1^ | 9.79x10^-6^ | 8.13x10^-1^ |
| rs16965644 | 17 | 1.22x10^-1^ | rs9853631 | 3 | 6.90x10^-1^ | 9.89x10^-6^ | NA |
| rs245082 | 5 | 9.20x10^-2^ | rs2013275 | 21 | 4.05x10^-1^ | 9.90x10^-6^ | NA |

SNP: single nucleotide polymorphism; Chr: chromosome; SNPA Pvalue: level of nominal statistical significance for single marker association with coronary artery disease for SNP A; SNPB Pvalue: level of nominal statistical significance for single marker association with coronary artery disease for SNP B; BHF-FHS: British Heart Foundation Family Heart Study; MIGen: Myocardial Infarction Genetics Consortium; Int Pvalue BHF-FHS: interaction P value in BHF-FHS; Int Pvalue MIGen: interaction P value in MIGen; N/A: replication not available.

**Supplementary Table D. Top SNP-SNP interactions between the 52 independent CAD-associated SNPs**

| SNPA | Chr A | SNPA Pvalue | SNP B | ChrB | SNPB Pvalue | Int. Pvalue WTCCC |
| --- | --- | --- | --- | --- | --- | --- |
| rs1412444 | 10 | 1.03X10^-01^ | rs515135 | 2 | 6.24X10^-01^ | 2.18X10^-03^ |
| rs17465637 | 1 | 2.83X10^-05^ | rs12526453 | 6 | 3.76X10^-02^ | 3.03X10^-03^ |
| rs12190287 | 6 | 7.91X10^-03^ | rs1561198 | 2 | 1.95X10^-02^ | 3.30X10^-03^ |
| rs2505083 | 10 | 8.70X10^-02^ | rs2075650 | 19 | 2.32X10^-01^ | 3.34X10^-03^ |
| rs1878406 | 4 | 5.13X10^-03^ | rs3184504 | 12 | 2.58X10^-02^ | 3.49X10^-03^ |
| rs3184504 | 12 | 2.58X10^-02^ | rs17114036 | 1 | 4.02X10^-02^ | 3.97X10^-03^ |
| rs579459 | 9 | 1.49X10^-03^ | rs12526453 | 6 | 3.76X10^-02^ | 4.25X10^-03^ |
| rs2048327 | 6 | 5.66X10^-04^ | rs2895811 | 14 | 3.40X10^-01^ | 4.47X10^-03^ |
| rs579459 | 9 | 1.49X10^-03^ | rs9369640 | 6 | 3.15X10^-02^ | 4.70X10^-03^ |
| rs501120 | 10 | 5.80X10^-04^ | rs12190287 | 6 | 7.91X10^-03^ | 5.30X10^-03^ |
| rs17465637 | 1 | 2.83X10^-05^ | rs9369640 | 6 | 3.15X10^-02^ | 5.93X10^-03^ |
| rs1878406 | 4 | 5.13X10^-03^ | rs2895811 | 14 | 3.40X10^-01^ | 6.60X10^-03^ |
| rs273909 | 5 | 1.77X10^-01^ | rs974819 | 11 | 7.31X10^-01^ | 7.55X10^-03^ |
| rs12190287 | 6 | 7.91X10^-03^ | rs12936587 | 17 | 9.14X10^-02^ | 8.86X10^-03^ |
| rs599839 | 1 | 1.68X10^-05^ | rs3184504 | 12 | 2.58X10^-02^ | 1.11X10^-02^ |
| rs46522 | 17 | 3.51X10^-03^ | rs2895811 | 14 | 3.40X10^-01^ | 1.15X10^-02^ |
| rs11206510 | 1 | 7.27X10^-04^ | rs579459 | 9 | 1.49X10^-03^ | 1.22X10^-02^ |
| rs9818870 | 3 | 6.59X10^-02^ | rs9515203 | 13 | 1.69X10^-01^ | 1.44X10^-02^ |
| rs3798220 | 6 | 6.83X10^-06^ | rs46522 | 17 | 3.51X10^-03^ | 1.65X10^-02^ |
| rs2954029 | 8 | 1.24X10^-04^ | rs3825807 | 15 | 3.79X10^-04^ | 1.67X10^-02^ |
| rs579459 | 9 | 1.49X10^-03^ | rs6544713 | 2 | 1.97X10^-02^ | 1.72X10^-02^ |
| rs3184504 | 12 | 2.58X10^-02^ | rs9818870 | 3 | 6.59X10^-02^ | 1.83X10^-02^ |
| rs9818870 | 3 | 6.59X10^-02^ | rs1412444 | 10 | 1.03X10^-01^ | 1.89X10^-02^ |
| rs46522 | 17 | 3.51X10^-03^ | rs12190287 | 6 | 7.91X10^-03^ | 1.91X10^-02^ |
| rs11206510 | 1 | 7.27X10^-04^ | rs273909 | 5 | 1.77X10^-01^ | 2.13X10^-02^ |
| rs501120 | 10 | 5.80X10^-04^ | rs9319428 | 13 | 1.99X10^-01^ | 2.35X10^-02^ |
| rs2023938 | 7 | 9.30X10^-02^ | rs9515203 | 13 | 1.69X10^-01^ | 2.36X10^-02^ |
| rs3184504 | 12 | 2.58X10^-02^ | rs10953541 | 7 | 3.42X10^-01^ | 2.55X10^-02^ |
| rs10947789 | 6 | 1.81X10^-01^ | rs4773144 | 13 | 2.15X10^-01^ | 2.63X10-^02^ |
| rs11203042 | 10 | 4.53X10^-02^ | rs2075650 | 19 | 2.32X10^-01^ | 2.75X10^-02^ |

SNP: single nucleotide polymorphism; Chr: chromosome; SNPA Pvalue: level of nominal statistical significance for single marker association with coronary artery disease for SNP A; SNPB Pvalue: level of nominal statistical significance for single marker association with coronary artery disease for SNP B; WTCCC: Wellcome Trust Cases Control Consortium.
